# The Brief Case: Knee joint infection caused by *Neisseria mucosa*

**DOI:** 10.1128/jcm.00717-25

**Published:** 2026-01-14

**Authors:** Cuiying Zheng, Minghui Song, Yumei Guo, Jiahao Hao, Shufang Liu, Jiaqing Ye, Weili Gao, Hong Zhang, Zhongjun Feng, Huifen Zuo, Zhenjun Zhao, Lijie Zhang

**Affiliations:** 1 Department of Laboratory Medicine, Hebei Medical University Third Hospital 74725, Shijiazhuang, Hebei, China; 2 Department of Laboratory Medicine, Hebei Key Laboratory of Intractable Pathogens, Shijiazhuang Center for Disease Control and Prevention 117967, Shijiazhuang, Hebei, China; 3 The First Hospital of Yongnian District, Handan, Hebei, China; 4 Department of Laboratory Medicine, Hebei Yiling Hospital 725664, Shijiazhuang, Hebei, China; Endeavor Health, Evanston, Illinois, USA

**Keywords:** *Neisseria mucosa*, knee joint infection, bacterial identification, 16S rRNA

## CASE

A 69-year-old woman presented with a history of hypertension, ischemic stroke, cardiac arrhythmia, hyperthyroidism, and coronary artery stenting but no history of immunosuppressive therapy. Three months prior, she was diagnosed with diabetes mellitus and initially treated with oral metformin for glycemic control; 1 month before admission, she discontinued metformin due to recurrent hypoglycemic episodes. Over the past 2 months, her right knee symptoms gradually worsened. She received initial treatment at a community hospital (oral analgesics and intra-articular sodium hyaluronate injections), but her condition did not improve. She was subsequently referred to a county-level integrated hospital. Imaging studies (MRI and X-ray) showed right knee osteoarthritis with purulent synovitis. Symptomatic anti-inflammatory and analgesic therapy was given, but without significant improvement. For further evaluation, the patient was referred to a municipal hospital. Joint fluid was aspirated and inoculated into aerobic, anaerobic, and fungal culture bottles. The aerobic culture grew *Staphylococcus aureus*, while the anaerobic and fungal cultures were negative. Antimicrobial susceptibility testing showed the isolate was sensitive to erythromycin and clindamycin. Based on these results, intravenous clindamycin was given for 14 days, but her symptoms did not improve. Three days after stopping the antibiotic, the patient was transferred to our Department of Orthopedics.

**Fig 1:**
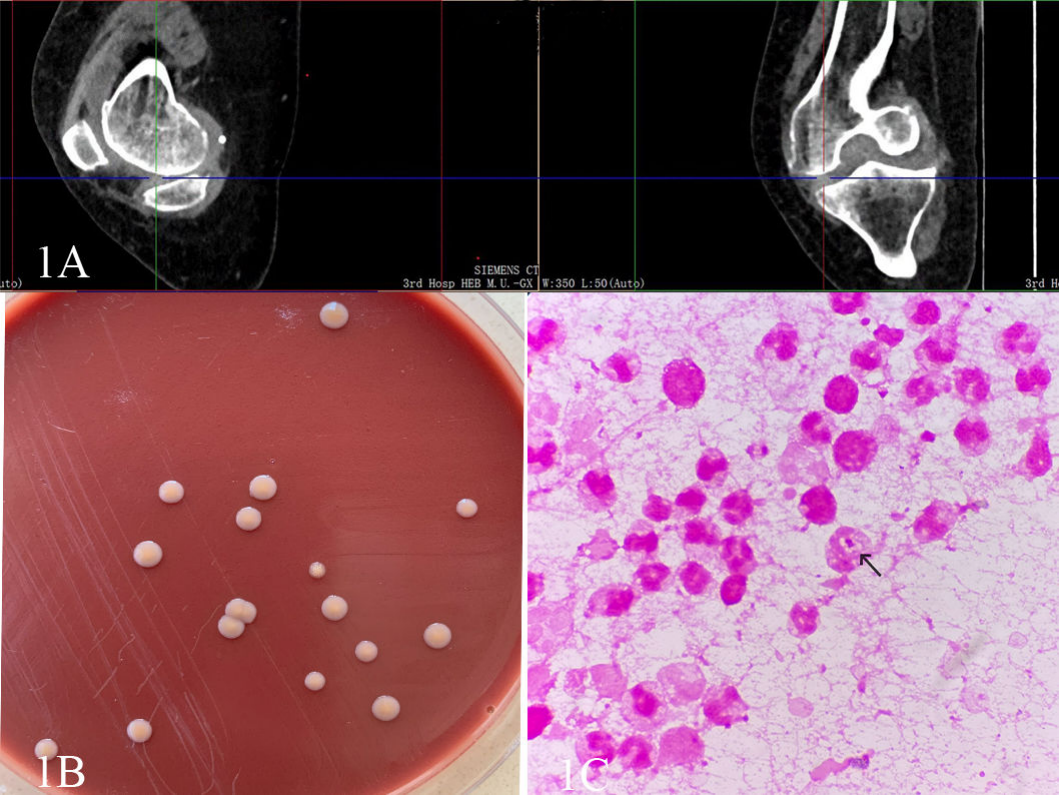
(**A**) The crosshair indicates a high-signal fluid in the joint cavity on the CT image. (**B**) Colony morphology of *N. mucosa* after 3 days of culture on chocolate agar. (**C**) The Gram staining of the joint fluid revealed leukocytes phagocytizing *N. mucosa* (×1,000).

Upon admission, the physical examination revealed swelling and hypertrophy of the right knee joint with a limited range of motion. Positive findings were noted in the patellar float test, patellar grind test, and tenderness test. Laboratory results showed a blood glucose level of 6.52 mmol/L, an erythrocyte sedimentation rate of 89.00 mm/h, and a high-sensitivity C-reactive protein level of 58.85 mg/L. The synovial fluid analysis indicated a red blood cell count of 100,000 × 10^6/L and a white blood cell count of 28,891 × 10^6/L. Elevations in all of these inflammatory indicators suggest infection in the knee joint. CT of the right knee demonstrated high-signal fluid in the joint cavity, indicative of joint effusion (Fig. 1A). Based on these findings, the patient was diagnosed with right knee osteoarthritis, joint effusion, and tears of the medial and lateral menisci.

In our clinic, under strict aseptic technique, synovial fluid from the right knee was aspirated and inoculated into aerobic and anaerobic culture bottles. The aerobic bottle became positive and was subcultured onto chocolate agar. After approximately 48 h of incubation, a few colonies appeared on the agar. These colonies were smooth, moist, non-hemolytic, creamy, and round and produced a pale yellow pigment (Fig. 1B). Gram staining of the colonies revealed gram-negative coccobacilli. Biochemical testing with a VITEK 2 NH card (BioMérieux, France) showed that the isolate fermented glucose and sucrose and was nitrate reduction-negative. Matrix-assisted laser desorption/ionization-time-of-flight (MALDI-TOF) mass spectrometry (Bruker Autoflex speed) identified the isolate as *Neisseria mucosa* with a confidence score of 2.248. To exclude contamination and confirm the pathogen, a second synovial fluid specimen was obtained on hospital day 3 after discussion between the microbiology laboratory and clinicians. Microscopy of the centrifuged fluid smear from this second aspirate showed abundant polymorphonuclear leukocytes (PMNs) and occasional neutrophils containing intracellular gram-negative diplococci (Fig. 1C). The isolate from this second culture was again identified by MALDI-TOF as *N. mucosa*. For definitive identification, 16S rRNA gene sequencing was performed using primers 1492R and 27F, yielding a 1401 bp sequence that was 100% identical to *N. mucosa* sequences in the GenBank database (GenBank accession PP713312). A third synovial fluid sample sent on day 8 also grew the same organism. Based on three independent cultures, microscopy showing phagocytosed gram-negative diplococci, and concordant MALDI-TOF and 16S sequencing results, *N. mucosa* was confirmed as the etiologic agent in this case. This is the first report of *N. mucosa* in a joint in China.

Antimicrobial susceptibility testing was performed by Etest on GC chocolate agar (Wenzhou Kangtai Biological) using Clinical and Laboratory Standards Institute (CLSI) breakpoints for *N. gonorrhoeae*. The minimum inhibitory concentrations were: ceftriaxone 0.25 mg/L, meropenem 0.25 mg/L, minocycline 4 mg/L, azithromycin 4 mg/L, rifampin 2 mg/L, and ciprofloxacin 1 mg/L. Based on these results, the patient was treated with intravenous ceftriaxone (2.0 g/daily). Given her multiple comorbidities and complex condition, a multidisciplinary team decided to defer surgical intervention on the knee. The patient was also given supportive treatments, including Dengzhan Shumai capsulesa, a traditional remedy for improving circulation, though not directly related to infection management, and her diabetes therapy was changed from metformin to vildagliptin (100 mg/daily). After 21 days of treatment, the patient was stable and discharged. She has remained free of recurrence on follow-up to date.

## DISCUSSION

The genus *Neisseria* is a group of gram-negative diplococci, which is mostly arranged in kidney-shaped pairs. These bacteria primarily colonize the oropharynx, nasopharynx, gastrointestinal tract, and urogenital tract of humans or animals. The genus *Neisseria* currently includes 34 species, most of which are commensal inhabitants of mucosal surfaces in humans and animals, including the oral cavity (1). *Neisseria* species isolated from humans include the *N. gonorrhoeae*, *N. meningitidis*, and several commensals, such as *N. cinerea*, *N. flavescens*, *N. lactamica*, *N. mucosa*, *N. polysaccharea*, *N. sicca*, *N. subflava*, and *N. elongata* (2). Apart from *N. meningitidis* and *N. gonorrhoeae*, which are highly pathogenic, the other *Neisseria* species rarely cause human infection. Clinical cases due to these organisms are relatively uncommon and may be overlooked; when infections do occur, species-level identification and antimicrobial susceptibility testing are crucial (3, 4).

We reviewed 23 case reports of *N. mucosa* infection published between 1985 and 2024. The affected patients ranged widely in age, from infants to the elderly, indicating that individuals of any age can be infected, although older patients and those with underlying diseases appear more susceptible. The reported infection sites varied and included endocarditis (eight cases, 35%) (3, 5–10), peritonitis (four cases, 17%) (11–14), and arthritis (one case, 4%) (15), as well as other sites, consistent with the colonization sites of *N. mucosa*, such as the respiratory tract and oral cavity. Additionally, underlying diseases or immunodeficiency (e.g., chronic neutropenia [16], systemic lupus erythematosus [7], IgA nephropathy [11], etc.) and various invasive procedures (e.g., cesarean section [17], tongue piercing [3], and ocular penetration [18]) were identified as high-risk factors for *N. mucosa* infection.

Identification of commensal *Neisseria* species is challenging. Biochemical testing alone has significant limitations, as it relies on carbohydrate utilization profiles, such as sugar fermentation tests, that often overlap among species. For example, *N. mucosa*, *N. sicca*, and *N. subflava* all ferment glucose and maltose, which can lead to misidentification of *N. mucosa* as one of the other species (19). MALDI-TOF mass spectrometry is rapid but relies on protein mass spectral patterns, which may not reliably distinguish genetically similar organisms (for example, differentiating *Escherichia coli* from Shigella or among *Neisseria* species), potentially causing confusion between *N. mucosa* and *N. meningitidis* or *N. cinerea* (20). In this case, MALDI-TOF identified the isolate as *N. mucosa*, but given the limitations of this technique, we performed 16S rRNA sequencing (which showed 100% identity with *N. mucosa* in GenBank) to resolve the taxonomic ambiguity. The concordant results of biochemical testing, MALDI-TOF, and 16S rRNA sequencing confirmed the organism as *N. mucosa*, highlighting the importance of genotypic identification for such bacteria.

The initial synovial fluid sample from the municipal hospital grew *Staphylococcus aureus*. However, *S. aureus* is a facultative anaerobe capable of growing under both aerobic and anaerobic conditions, whereas in this case, it was isolated only in the aerobic culture (the anaerobic culture remained negative), and 14 days of clindamycin therapy was ineffective. These findings suggested that the *S. aureus* was likely a skin contaminant introduced during aspiration rather than the true pathogen. In contrast, three consecutive synovial fluid samples from our hospital all grew *N. mucosa*, and microscopic examination consistently showed neutrophils containing gram-negative diplococci, clearly implicating *N. mucosa* as the pathogen responsible for the knee infection. Moreover, the patient had multiple chronic diseases and a history of invasive procedures, all of which are high-risk factors for *N. mucosa* infection. This case not only expands the known clinical spectrum of *N. mucosa* infection but also provides a reference for diagnosis and treatment of similar cases.

Antimicrobial susceptibility testing showed that the isolate was sensitive to ceftriaxone, consistent with reports that most *N. mucosa* strains are susceptible to β-lactam antibiotics (4, 6). Case reviews indicate that ceftriaxone achieves clinical cure in over 90% of invasive *N. mucosa* infections. However, in our case, the isolate had relatively high MICs to azithromycin and minocycline possibly related to efflux pumps or ribosomal modification mechanisms commonly found in commensal *Neisseria* (20). Since there are currently no standardized susceptibility testing methods for *N. mucosa*, the literature suggests using standard methods for fastidious *Neisseria* (GC chocolate agar with 5–7% CO₂, broth or agar dilution, or Etest) (21). We followed these guidelines by using the Etest for susceptibility testing. Specifically, we prepared a suspension of the isolate to a 0.5 McFarland standard, inoculated it onto GC chocolate agar, and incubated at 37°C with 5–7% CO₂ for 18–24 h, reporting MICs in mg/L. We referred to CLSI breakpoints for *N. gonorrhoeae*, but due to interspecies differences in resistance gene expression, interpretation may be biased. Whether it is appropriate to apply CLSI breakpoints for *N. gonorrhoeae* or *N. meningitidis* to *N. mucosa* requires further study and accumulation of additional case data to establish clearer treatment guidelines.

As a normal member of the skin and mucosal microbiota, *N. mucosa* rarely causes invasive opportunistic infections in immunocompetent hosts. This case exemplifies its rare clinical pathogenic potential in immunocompromised individuals. The patient had several underlying conditions, including hypertension, diabetes, and arrhythmias, which may have led to impaired immune function. In this particular context, *N. mucosa* took advantage of the weakened immune system and became the pathogen responsible for the joint infection. This article reports the first case in China of purulent knee arthritis caused by *N. mucosa*, and further research is needed regarding its pathogenic mechanisms, modes of transmission, and treatment options. This case not only provides valuable clinical data on *N. mucosa* but also emphasizes the importance of paying sufficient attention to these microorganisms, which are typically regarded as part of the normal flora and low-pathogenicity organisms. Timely and efficient laboratory testing and diagnosis are crucial for the early detection of *N. mucosa* infections, provision of appropriate treatment, enhancement of the awareness of microbiologists and clinicians regarding this organism, and provision for reference for clinical diagnosis and treatment of *N. mucosa* infection in the knee joint.

## SELF-ASSESSMENT QUESTIONS

1. In this patient with septic arthritis of the knee, which finding most strongly indicates that *Neisseria mucosa* is the true pathogen rather than a contaminant?
  - The same organism was isolated from synovial fluid on two separate cultures, and Gram stain of the fluid showed neutrophils containing gram-negative diplococci.
  - *N. mucosa* was isolated from synovial fluid cultures, but Gram stain revealed only inflammatory cells without visible bacteria, and repeat cultures were negative.
  - The organism grew in a single synovial fluid culture and was identified by MALDI-TOF MS.
  - *N. mucosa* was also found in the patient’s oropharyngeal flora on throat swab.
2. Which statement best describes the clinical significance of *Neisseria mucosa* as it relates to this case?
  - *N. mucosa* is an obligate human pathogen that frequently causes joint infections.
  - *N. mucosa* is part of the normal nasopharyngeal flora but can cause invasive infections in immunocompromised patients.
  - *N. mucosa* commonly causes mild throat infections in healthy adults.
  - *N. mucosa* is primarily a zoonotic organism acquired from animal contacts.
3. Which diagnostic approach most definitively confirms the identity of the unusual *Neisseria mucosa* isolate in this case?
  - Gram stain of synovial fluid and oxidase test on the isolate
  - MALDI-TOF MS identification of the isolate (without further testing)
  - MALDI-TOF MS identification, followed by 16S rRNA gene sequencing confirmation
  - Conventional biochemical testing using commercial identification panels

## ANSWERS TO SELF-ASSESSMENT QUESTIONS

1. In this patient with septic arthritis of the knee, which finding most strongly indicates that *Neisseria mucosa* is the true pathogen rather than a contaminant?
  - The same organism was isolated from synovial fluid on two separate cultures, and Gram stain of the fluid showed neutrophils containing gram-negative diplococci.
  - *N. mucosa* was isolated from synovial fluid cultures, but Gram stain revealed only inflammatory cells without visible bacteria, and repeat cultures were negative.
  - The organism grew in a single synovial fluid culture and was identified by MALDI-TOF MS.
  - *N. mucosa* was also found in the patient’s oropharyngeal flora on throat swab.

Answer: a. The same organism was isolated from synovial fluid on two separate cultures, and Gram stain of the fluid showed neutrophils containing gram-negative diplococci.

True infection of a sterile site is supported by reproducible isolation of the organism plus direct evidence of host response. In this case, *N. mucosa* was recovered from sterile synovial fluid twice, and Gram stain showed neutrophils phagocytosing gram-negative diplococci. Repeated positive cultures from synovial fluid and observation of intracellular organisms demonstrate active infection rather than contamination. In contrast, a single culture (option C) or finding in throat swab (option D) could reflect colonization, and isolation without phagocytosed bacteria and had negative repeat cultures (option B) is less definitive.

- Which statement best describes the clinical significance of *Neisseria mucosa* as it relates to this case?
  - *N. mucosa* is an obligate human pathogen that frequently causes joint infections.
  - *N. mucosa* is part of the normal nasopharyngeal flora but can cause invasive infections in immunocompromised patients.
  - *N. mucosa* commonly causes mild throat infections in healthy adults.
  - *N. mucosa* is primarily a zoonotic organism acquired from animal contacts.

Answer: b. *N. mucosa* is part of the normal nasopharyngeal flora but can cause invasive infections in immunocompromised patients.

*Neisseria mucosa* normally colonizes the oropharynx and upper respiratory tract and is usually nonpathogenic. It is not an obligate pathogen and rarely causes disease in healthy hosts. However, a systematic review of 23 global cases of *N. mucosa* infections in PubMed (1985–2024) has reported infections involving the meninges (1991), conjunctiva (1987), lungs (2011), heart valves (2014), peritoneal cavity (2016), and urinary tract (2024), particularly in immunocompromised individuals. Thus, statement B correctly highlights that a normally commensal organism can become invasive under host immunosuppression. Options A, C, and D are incorrect because *N. mucosa* is not commonly pathogenic, does not typically cause routine pharyngitis, and is not zoonotic.

- Which diagnostic approach most definitively confirms the identity of the unusual *Neisseria mucosa* isolate in this case?
  - Gram stain of synovial fluid and oxidase test on the isolate
  - MALDI-TOF MS identification of the isolate (without further testing)
  - MALDI-TOF MS identification, followed by 16S rRNA gene sequencing confirmation.
  - Conventional biochemical testing using commercial identification panels

Answer: c. MALDI-TOF MS identification, followed by 16S rRNA gene sequencing confirmation.

Modern microbiology labs use MALDI-TOF MS for rapid species identification, especially for uncommon or fastidious bacteria. However, when an unusual species is encountered, confirmatory 16S rRNA gene sequencing is recommended. Thus, combining MALDI-TOF MS with 16S sequencing (option C) provides the most definitive identification. Gram stain/oxidase (A) or conventional biochemicals (D) are slow and less specific, and MALDI-TOF alone (B) may need molecular confirmation for a rare pathogen.

**TAKE-HOME POINTS**

- The diagnosis of rare pathogens requires a multimodal laboratory confirmation: In this case, *N. mucosa* was identified through repeated synovial fluid cultures, microscopy showing phagocytosed gram-negative diplococci, MALDI-TOF MS, and 16S rRNA sequencing. These combined methods are critical to distinguishing true infection from contamination, particularly for low-virulence commensals like *N. mucosa*.
- Immunocompromised hosts are at risk for opportunistic infections: The patient’s multiple comorbidities highlight the importance of considering atypical pathogens in vulnerable populations. *N. mucosa*, typically a mucosal commensal, can cause invasive infections under compromised immunity.
- Treatment challenges and the need for standardized guidelines: Antibiotic selection relied on susceptibility testing interpreted via the CLSI guidelines for *N. gonorrhoeae*, as no specific standards exist for *N. mucosa*. This case underscores the necessity for broader antimicrobial susceptibility data and pathogen-specific protocols to optimize the management of rare infections.

## Data Availability

All data generated and analyzed during this study are included in this published article.
